# A ‘COMMON SENSE’ approach to geriatric patients in clinical practice

**DOI:** 10.4102/safp.v67i1.6027

**Published:** 2025-03-05

**Authors:** Lara S. Greenstein

**Affiliations:** 1 Department of Internal Medicine, Faculty of Health Sciences, University of the Witwatersrand, Johannesburg, South Africa; 2 Division of Geriatric Medicine, Faculty of Health Sciences, Helen Joseph Hospital, University of the Witwatersrand, Johannesburg, South Africa

**Keywords:** common sense, screening tools, geriatrics syndromes, comprehensive geriatric assessment, common geriatric conditions

## Abstract

South Africa has a critical shortage of geriatricians and a growing ageing population. Most geriatric patients are cared for by their primary care practitioner who may not have been trained in the care of the older adult. The comprehensive geriatric assessment (CGA) is the cornerstone of the geriatric consultation but can be time-consuming. By using a common sense approach to the geriatric patient, none of the important components of the CGA will be missed. The mnemonic ‘COMMON SENSE’ can be used as a tool to assist in identifying the common conditions that older adults experience, as well as highlight specific considerations that become increasingly important in this population. Many simple and time-effective screening tools are available to assist in diagnosing the geriatric syndromes which can be easily implemented in a busy primary care practice.

## Introduction

The number of people aged 65 years and above in South Africa is increasing and general practitioners are treating a growing number of older patients.^1,2^ Geriatric patients often have multiple underlying conditions, and because of this complexity, consulting with older adults may be daunting.^3^ There is a critical shortage of geriatricians in South Africa and the general practitioner often has to take on the role of overseer. By applying the mnemonic ‘COMMON SENSE’, which is being coined in this article (see Box 1), important considerations and common conditions affecting older adults are less likely to be missed. Awareness of these geriatric syndromes enables clinicians to utilise simple targeted screening tools to identify conditions that may not be immediately apparent, thereby optimising the quality of care provided to older patients. As only 15–20 min is allocate to a general practice consultation, not every condition can be assessed in this short time, and individualised care is necessary to decide which components to tackle in each appointment. An hour-long booking for a comprehensive geriatric assessment (CGA) would be ideal.

BOX 1 The components of COMMON SENSE.

**Table:** 

C	Comprehensive geriatric assessment
O_4_	Optimise Treatments Targets Function Preventative strategies
${\text{M}}_{5}^{8}$	Mind (depression, delirium and dementia) Mobility (falls and instability) Medications Multicomplexity (multimorbidity) Matters most (goals and care)
O_2_	Oops Incontinence Iatrogenesis – medication, procedure or diagnostic testing related
N	Nutrition
S	Syndromes The 5 Ms Incontinence Iatrogenesis Loneliness Frailty Sarcopenia Malnutrition
E	Environment – care needs
N	Networking and multidisciplinary teams
S	Safety (The *Older Persons Act*)
E	End of life care and advanced care planning

Note: Please see the full reference list of this article https://doi.org/10.4102/safp.v67i1.6027 for more information.

## The comprehensive geriatric assessment

**FIGURE 1:**
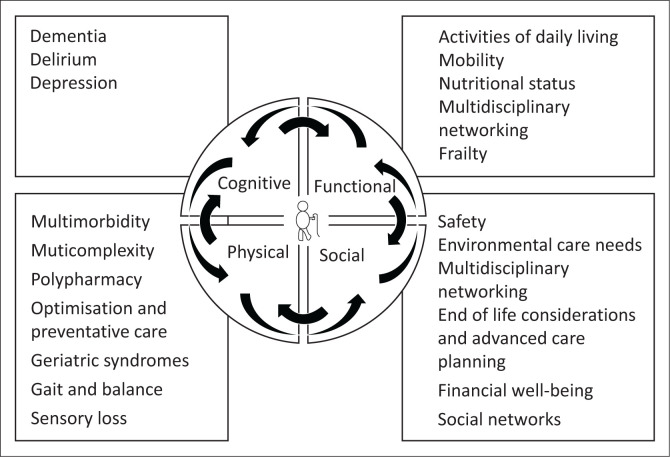
The comprehensive geriatric assessment with the COMMON SENSE components.

The CGA is the cornerstone of geriatric medicine, taking into account all the components of the COMMON SENSE mnemonic (see Figure 1). It enhances the well-established biopsychosocial model by emphasising the critical interplay of these three vital components, while integrating cognitive and functional assessments to provide a more comprehensive approach to patient care. The CGA is a multidimensional approach to care of the older person underscoring the need for a multidisciplinary team approach.^4^ The members of this team include all allied health professionals, often various medical practitioners, pharmacists and nursing staff. This framework facilitates the creation of a comprehensive problem list and the development of an individually tailored care plan. The care plans, along with the implemented interventions, should be regularly reviewed and adjusted as necessary. The CGA can be utilised in a variety of settings, both inpatient and outpatient. It is particularly valuable when a general practitioner is managing a frail older adult with multiple geriatric syndromes, during transitions in care settings and following hospital discharge.^4,5,6^ The remainder of this article will discuss the individual components of the CGA with a focus on the COMMON SENSE components and will discuss the most time and cost-effective screening tools to assess these common problems.

## The 5 Ms and the geriatric syndromes

Geriatric syndromes are common conditions that occur in older adults as a result of multiple contributing factors.^7^ Consequences of these syndromes are the loss of independence, deteriorating health, higher healthcare costs, the need for institutionalised care, morbidity and mortality.^7^ In 2017, the 5 Ms (see Box 1) were introduced to help those practitioners who care for older patients to identify common geriatric problems.^8^ The 5Ms take into account many of the geriatric syndromes including delirium, dementia, depression, falls and instability, polypharmacy, multimorbidity and goals of care.^8^ There are a few other syndromes, however, that deserve mention, including loneliness, frailty, sarcopenia, malnutrition, incontinence and iatrogenesis. Iatrogenesis comes in many forms and may result from medications, adverse drug events, or complications from procedures, which may be diagnostic or therapeutic. These syndromes are frequently underdiagnosed because of the complexities involved in managing older adults.

## Optimisation of the older adult

The main goals of successful ageing are to keep the older adult as functional as possible for as long as possible, and as this is a multidimensional concept, successful ageing incorporates all the CGA components including functional, cognitive, social and psychological well-being.^9^ One way to achieve this is to optimise medication and treatment regimes, optimise function, aim for appropriate therapeutic targets and make use of preventative strategies.

Functional status is the ability of the older adult to look after themselves and perform the activities of daily living required of them to live independently.^9^ Box 2^15,16,17^ shows both the basic and instrumental activities of daily living. If a person cannot perform instrumental activities of daily living safely by themselves, then they will require help – either at home or in a mid-care type setting. Inability to complete certain instrumental activities independently such as driving, administering medication and taking care of financial affairs can pose a physical or financial safety threat. Those who cannot perform basic activities of daily living independently, such as eating, toileting and transferring from a bed to a chair will require 24-h care either at home or in a frail-care setting.

BOX 2 Activities of daily living.

**Table:** 

**Basic activities of daily living** ^**15**^
Bathing
Dressing
Toileting
Transferring from a bed to a chair
Feeding
**Instrumental activities of daily living^16,17^**
Shopping
Phoning
Food preparation
Laundry
Transportation
Financial affairs
Administering own medication

Note: Please see the full reference list of this article https://doi.org/10.4102/safp.v67i1.6027 for more information.

Targets for common conditions such as type 2 diabetes mellitus and hypertension depend on the person’s functional and frailty status. While tighter control of both blood pressure and glucose measurements are recommended for healthy older adults who are able to complete all their own instrumental activities of daily living and who are not frail, systolic blood pressures of 150 mmHg^10^ and heamoglobin A1C (HbA1C) measurements of between 7% and 8.5%^11^ are acceptable for those patients who have multiple chronic long-term conditions, who are frail and who are nearing end of life.

Optimising medications is vitally important because inappropriate polypharmacy, taking more medications than are medically necessary or using medication that is ineffective, can lead to deleterious consequences such as adverse drug reactions, morbidity and mortality.^12^ Useful tools that can be used to assist with rationalising medications include the use of drug-interaction checkers, the Beers criteria and the STOPP/START criteria (see Table 1).^13,14^

**TABLE 1:** The simplest screening tools that can be implemented in primary care practice for the common geriatric syndromes.

Geriatric syndrome	Assessment tool	Interpretation	Sensitivity and specificity	References
Cognitive impairment (dementia)	Mini-cog If abnormal, perform a Folstein Mini-Mental examination or Montreal Cognitive Assessment test.	Give the patient three words to remember. Ask the patient to draw a clock and make the time ten past nine. Ask the patient to recall all three words. If all three words correct – pass If all three words incorrect – fail If one or two words correct and clock is normal – pass If one or two words correct and clock is abnormal – fail	Sensitivity: 99% Specificity: 93%	^24,25^
Depression	2-item Geriatric Depression Scale test If abnormal – perform the 15-item short form geriatric Depression Scale	An answer of yes to either of: During the past month, have you Being down, depressed or hopeless Having little interest or pleasure in doing things?	Sensitivity: 91.8% Specificity: 67.7%	^26^
Polypharmacy	Use a drug-interaction checker, the Beers criteria or STOPP/START criteria	The Beers criteria is a guideline used to identify potentially inappropriate medicines that should be avoided in older adults. The STOPP/START criteria identify potentially inappropriate prescriptions (STOPP) and recommend appropriate medications (START).	-	^13,14^
Frailty	The Clinical Frail Scale	1 point each for the following: Fatigue – exhaustion in the previous 2 weeks Resistance – the inability to walk up one flight of stairs Ambulation – the inability to walk one block Illnesses – ≥ 5 Loss of weight ≥ 5% in the past year Scoring: 0 = robust; 1–2 = pre-frail; ≥ 3 = frail	Sensitivity: 88.0% Specificity: 85.7%	^27,28^
Malnutrition	Nestle Mini Nutritional Assessment	Questions asking about food intake, weight loss, mobility, psychological stress and neuropsychological problems. The body mass index (BMI) is measured and if unavailable, the calf circumference can be used. Scoring < 7 = malnourished; 8–11 = at risk; ≥ 12 = normal.	Sensitivity: 96% Specificity: 98%	^29^
	The Simplified Nutritional Appetite Questionnaire (SNAQ)	Four questions asking about appetite, satiation, food taste and number of meals per day. Score ≤ 14 indicates a high risk for at least 5% weight loss in 6 months.	Sensitivity: 69.2% Specificity: 61.3%	^30^
Gait/balance abnormalities	Timed-Up-and-Go (TUG)	The patient must be timed while rising from a chair, walking three metres, turning around, walking back and sitting down. They can use their usual walking aids. A time of < 10 s is normal, while > 14 s indicates a high risk of falling.	-	^23^
Falls	Timed-Up-and-Go with or without a fall risk assessment	Ask each patient every year if they have fallen. One fall with no injuries → perform the TUG, and if normal, no intervention. If the TUG is abnormal or there have been two or more falls or a fall resulting in a serious injury → full falls assessment including history, examination and special investigations.	Sensitivity: 0.31 (95% CI: 0.13–0.57) Specificity: 0.74 (95% CI: 0.52–0.88)	^31,32^
Incontinence	3IQ test	Have you leaked urine in the past 3 months? If yes: with activity – stress incontinence with urgency – urge incontinence both – mixed incontinence neither – another cause	Sensitivity: 75% Specificity: 77%	^33^
Sarcopenia	Sarc-F Questionnaire	A number of questions each scoring a maximum of 2 points for strength, assistance in walking, rising from a chair, climbing stairs and falling. ≥ 4 indicates sarcopenia.	Sensitivity: 28.9% – 55.3% Specificity: 68.9% – 88.9%	^34,35^

Note: Please see the full reference list of this article https://doi.org/10.4102/safp.v67i1.6027 for more information.

CI, confidence interval; IQ, incontinence questions; Sarc-F, Strength, Assistance with walking, Rising from a chair, Climbing stairs, and Falls.

Other optimisation strategies include vaccinations and the screening for osteoporosis and common malignancies such as those arising from the prostate, breast and colon. The decision to undergo certain screening tests depends on whether the benefits outweigh the risks, personal preferences and the likelihood that the patient will live long enough to experience the benefits of treating any conditions that are diagnosed.

## Social and care needs of the older adult

Choosing the most appropriate care setting is dependent on the cognitive and functional status of the older person. Other factors that need to be taken into account include the availability of social support networks and finances. Frail-care facilities can be expensive and the waiting times for admission can be long. A poor support system can result in loneliness, which itself is a risk factor for poor health.^18^ Older adults constitute a vulnerable population and are therefore safeguarded by the *Older Persons Act*,^19^ which mandates the reporting of any suspected abuse or neglect against an older person.^20^

Advance care planning and end of life decisions become increasingly more important to discuss as the older adult becomes frailer. Discussions around healthcare proxies, expectations at the end of life and living wills cannot be adequately addressed in the last few minutes of a consultation, and a healthcare practitioner who has an established and trusted relationship with the patient is best suited to initiate these discussions.^21^ Currently, there is no law in South Africa to validate a living will; however, there is an ethical expectation from the patient who has a living will that their wishes will be followed.^22^

## Simple screening tools

A number of screening tools and toolkits are available to assist in best diagnosing the various geriatric syndromes. As a result of both time and resource constraints in the primary care setting, a simple screening of the major geriatric syndromes would be sufficient to screen for the majority of these syndromes (see Table 1). Those who are flagged can then be interrogated in more depth or referred on to the appropriate clinician or allied healthcare worker.^6^ The CGA itself can take up to 2 hours to complete and this is usually not possible during the course of a single short consultation.

For certain syndromes such as hearing impairment and poor vision, a simple yes or no question asking if there are any problems is adequate. Screening for every syndrome is sometimes not achievable in the course of a consultation, and for a busy practice, the most important syndromes to ask about would be falls, memory problems and to perform a medication review. The Timed-Up-and-Go (TUG) takes a few seconds and gives a wealth of information.^23^ Difficulty in getting up from the chair may indicate proximal muscle weakness; gait abnormalities can be seen and characterised when the patient walks the three metres, and both static and dynamic balance problems can be detected. Screening for cognition can be performed by administering the simple mini-cog, which provides information on many aspects of cognition.^24^ Both immediate and delayed recall are tested with the three words and executive function, planning and sequencing with the clock drawing test.

## Conclusion

Geriatric syndromes are common and often difficult to diagnose. Assessment tools can be a simple and cost-effective means to diagnose these syndromes. In addition to the standard history and examination, using a simple mnemonic ‘COMMON SENSE’ allows the primary care physician to remember the important components of the geriatric assessment.
